# The impact of nutritional quality and gut bacteria on the fitness of *Bactrocera minax* (Diptera: Tephritidae)

**DOI:** 10.1098/rsos.180237

**Published:** 2018-07-11

**Authors:** Awawing A. Andongma, Lun Wan, Xue-ping Dong, Mazarin Akami, Jin He, Anthony R. Clarke, Chang-Ying Niu

**Affiliations:** 1 College of Plant Science & Technology, Huazhong Agricultural University, Wuhan 430070, People's Republic of China; 2 State Key Laboratory of Agricultural Microbiology, Huazhong Agricultural University, Wuhan 430070, People's Republic of China; 3 Hubei Center for Disease Control and Prevention, Zhuodaoquanbei Road 6, Wuhan, 430079, People's Republic of China; 4 School of Earth, Environmental, and Biological Sciences, Queensland University of Technology, Brisbane, 4001 Queensland, Australia

**Keywords:** Tephritidae, diet quality, symbiotic bacteria, Dacinae

## Abstract

To examine how nutritional quality and resident gut bacteria interplay in improving the fitness of an oligophagous fruit fly, *Bactrocera minax*, artificial sucrose diets and full diets (sucrose, tryptone and yeast extract) were fed to flies with and without antibiotic supplementation. Furthermore, *Klebsiella oxytoca* and *Citrobacter freundii* were supplemented to sucrose-only diets. Flies were maintained in the laboratory and the fitness parameters, male and female longevity, number of copulations and female fecundity, were recorded. Full diet without bacterial depletion significantly increased fecundity and copulation. In the absence of gut bacteria, flies fed with full diets had significantly decreased mean fecundity and copulation rate. Flies that were fed with sucrose diet had a very low copulation rate and produced no eggs. Diet type and the presence of bacteria did not have any effect on the average longevity of male and female flies. Bacterial supplementation in sucrose diets did not improve any of the measured parameters. The results demonstrate that gut bacteria interact with diet to influence mating and reproduction in *B. minax*. Symbiotic bacteria significantly and positively impact reproduction in *B. minax*; however, their impact can only be fully realized when the flies are fed with a nutritionally complete diet.

## 1. Introduction

Many insect species are able to survive and reproduce despite feeding on what are nutritionally poor diets. For example, aphids live on plant sap which is of low nutritional quality [1], while termites live on a non-degradable cellulose diet [2]. That these organisms, and numerous other examples, are nutritionally successful is considered to be the result of diet modification by symbiotic bacteria that these insects harbour [3].

The relationship between insects and their symbiotic bacteria dates back to the most primitive insects [4] and has had major impacts on insect evolution [3,5,6]. Resident bacteria have been isolated from the digestive tracts of many different insects, and the important roles they play in insect nutrition were widely accepted [7–9]. In some cases, the symbiont and its host insect have developed a very intimate relationship, where they depend primarily on each other for nutrition [1]. In other cases, the role symbiotic bacteria play in nutrition is less apparent, except when the host insect is nutritionally stressed. For example, *Hamiltonella* sp. infested white flies grew better than uninfected flies when reared with low nitrogen diets. However, no significant differences were observed when both groups were reared on a standard diet [10].

Fruit flies of the family Tephritidae (Insecta: Diptera) have close relationships with symbiotic bacteria [11,12], and gut bacteria form an essential part of the nutritional ecology of fruit flies [13,14]. Commonly, gut microbial fauna synthesize amino acids which do not otherwise occur in the diet, and this can lead to increased protein synthesis and female fecundity. Such results have been reported for the olive fruit fly, *Bactrocera oleae* (Rossi) [13,15], the apple maggot fly, *Rhagoletis pomonella* (Walsh) [16] and the Mediterranean fruit fly, *Ceratitis capitata* (Wiedemann) [17,18].

While there are numerous papers supporting the link between bacteria and fruit fly nutrition, the field is not without ambiguity, or the need for further species-specific research. For example, Drew *et al*. [19], determined that the fecundity of Queensland fruit fly, *Bactrocera tryoni* (Froggatt), did not differ when fed a diet of bacteria, sugar and water versus a diet of brewer's yeast, sugar and water. This author, therefore, concluded that bacteria act as a natural food source for these fruit flies. However, Meats *et al*. [20] did not record any differential fecundity effects of bacteria feeding to *B. tryoni*. Such mixed results reinforce a point that for many fruit flies it is still unclear if bacteria act as a direct dietary source, act as modifiers of other diet, or play one or both roles but with an impact only being apparent if the host insect has, in some way, a dietary stress.

*Bactrocera minax* (Enderlein), known for breeding only on a citrus host plant, causes serious economic damage in southern/central China, Bhutan and neighbouring regions [21,22]. In support of pest management, significant research effort is currently being applied to understanding *B. minax* biology and ecology, including its bacterial relationships [23–25]. A previous study [26] on the impact of isolated bacterial strains on the fitness of the *B. minax* fruit fly had complex results which varied depending on the bacterial strain and the fitness parameters measured. In general, the incorporation of *Pseudomonas dispersa*, *Klebsiella pneumonia* and *Citrobacter braakii* into full diets decreased the longevity of male and female flies and improved female fecundity. However, only *C. braakii* supplementation significantly increased the number of observed matings. Though this study informs us that bacteria can influence fitness parameters of *B. minax*, it still remains unclear if these impacts are a result of the supplemented bacteria acting as additional dietary components, whether the bacteria modify the quality of the base diet, or if it is due to an interaction of both effects. It should be noted here that members of the genera *Citrobacter* and *Klebsiella* dominate the gut microbial fauna of many tephritid species including *C. capitata* [27], the oriental fruit fly, *Bactrocera dorsalis* (Hendel) [28] and *B. minax* [24].

To determine the mechanism(s) of how variable diet and gut bacteria interact to modify fitness of *B. minax*, we carried out two types of bacterial modification (antibiotic knock-down and bacterial supplementation) with two diet types (sucrose-only and sucrose + protein (= ‘full’) diets) and fed to adult flies. Our recorded fitness measures were, as for [26], longevity, fecundity and mating number. We hypothesized that the presence of gut bacteria will supplement the nutrition provided by both the sugar and ‘full’ diets, resulting in an overall improvement in fitness; while the absence of the gut bacteria would negatively impact the fitness parameters.

## 2. Material and methods

### 2.1. Insects and rearing conditions

All insects were collected from infested fruit from the Yichang district (30.69° N, 111.29° E), Hubei Province, China, and returned to laboratory at Huazhong Agricultural University, Wuhan. Third-instar larvae were allowed to pupate into sterilized soil in the laboratory, with subsequent adult emergence into sterilized mesh cages supplied with sugar and water. The laboratory conditions were as follows: temperature 27 ± 1°C, RH 70%, 12 L: 12 D. A total of 20 (10 males and 10 females) flies were held in each cage (45 × 30 × 30 cm) and each treatment was replicated three times. Replication number was restricted by the need to rear adults from naturally infested fruit, as it is not currently possible to laboratory culture this fly over multiple generations as it is univoltine with an obligate six-month diapause [21].

### 2.2. Experiment 1, interaction of diet and antibiotic treatments

Adult flies were used three days after emergence. Experimental flies were fed with double-distilled water-based sucrose or ‘full’ diets, with or without antibiotics. Flies fed with dissolved sucrose diets had no source of protein. By contrast, the ‘full’ diet contained sucrose, trypton and yeast extract (Beijing Shuangxuan microbial culture medium product factory, Beijing, China), which supplied flies with amino acids, minerals and carbohydrates [29]: diet details are provided in table 1. When antibiotics (10 µg ml^−1^ ciprofloxacin and 200 µg ml^−1^ piperacillin) were added to diet, it was first diluted in sterilized, double-distilled (DD) water and then filter-sterilized. The antibiotics used in this experiment have previously been used to suppress the gut bacteria in *C. capitata* and *B. oleae* [17,30–32]; however, antimicrobial susceptibility tests were carried out to confirm that bacteria strains isolated from the gut of the *B. minax* used had not developed resistance against these antibiotics (data available upon request from the senior author). After preparation, experimental diets were refrigerated at −20°C and used within one month. Diets were pipetted into Petri dishes containing a filter paper and offered and changed on a daily basis.

**Table 1.** Composition of four experimental diets fed to *B. minax* adults (values in µg ml^−1^ of double-distilled water). The yeast extract used is from Beijing Shuangxuan microbial culture medium product factory, Beijing, China.

	sucrose	sucrose + antibiotics	full diet	full diet + antibiotics
sucrose	200 000	200 000	200 000	200 000
yeast extract			40 000	40 000
trypton			60 000	60 000
antibiotics				
gentamicin		10		10
piperacillin		200		200

### 2.3. Experiment 2, bacterial supplementation of sugar-only diets

In a separate experiment, we further investigated if specific bacteria isolates could improve on the low nutritional quality of sugar diets. Newly emerged flies were fed with antibiotics and sucrose diets for 4 days, then a concentration of 10^8^ CFU ml^−1^ *Klebsiella oxytoca* and *Citrobacter freundii* previously isolated from the gut of *B. minax* were separately supplemented in the sucrose diet. Isolation and identification of these bacteria strains was carried out as reported by [26]. These sequences have been submitted to the NCBI GenBank under the reference number KF145191 and KF145194. Other than the diet, all other aspects of this trial were identical to the first.

### 2.4. Population fitness assessment

#### 2.4.1. Longevity

The number and sex of dead insects per cage were determined each day until all flies died. The value used in the analysis was the mean longevity in days for each sex and each cage.

#### 2.4.2. Mating

*Bactrocera minax* mates on the host plant, with peak mating during the middle of the day [25,33]. To mimic this, experimental cages were provided with a small potted valencia orange plant (*Citrus c*. * *sinesis* (L)). Ten sexually matured virgin males and females were kept within a cage and the number of mating pairs was recorded from 11.00 to 18.00 each day for 15 consecutive days for each of the three replicate treatment groups. The summed number of pairs over 15 days was the replicate data used for analysis.

#### 2.4.3. Fecundity

Orange fruits were placed in experimental cages as oviposition substrates. Fruits were changed every three days and the number of eggs per fruit was counted following fruit dissection. The average fecundity per female was calculated by dividing the total number of eggs collected from each cage at the end of the experiment by the average number of females present in each cage during the fecundity period.

### 2.5. Statistical analysis

For experiment 1, a two-way ANOVA was performed to test the effects of diet type and antibiotics on the longevity (male and female), and copulation number. The independent variables were diet (sugar diet versus full diet) and antibiotic treatment (present versus absent). The dependent variables were mean male and female longevity per replicate and total number of matings per replicate. For fecundity, flies on the sugar-only diet, with or without antibiotics, produced no eggs, so the effect of diet was not statistically tested. The effect of antibiotic treatment on egg production of flies on the full diet (Expt 1), and bacterial supplementation in sugar diets (Expt 2) were tested using one-way ANOVA. Levene's test was used to assess homogeneity of all the datasets and only the effects of supplemented bacteria on male longevity required log transformations. Multiple comparisons between treatments were based on Tukey's post hoc tests. A *p*-value ≤ 0.05 was taken as significant. All datasets were analysed using SPSS 16.0 statistical software.

## 3. Results

### 3.1. Longevity

**Figure 1.**
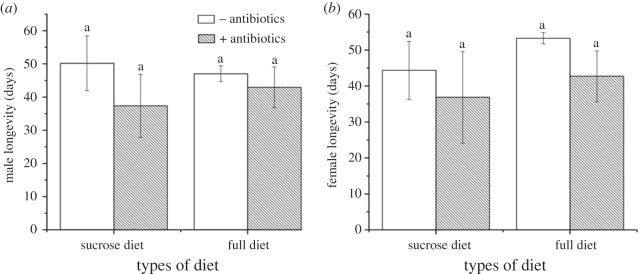
The effects of diet type and antibiotic treatment (10 µg ml^−1^ ciprofloxacin and 200 µg ml^−1^ piperacillin) on the mean (s.d.) longevity in days of male (*a*) and female (*b*) *Bactrocera minax*. Unshaded bars are diets with no antibiotics added; shaded bars are diets with antibiotics added. Columns with the same letters are not significantly different after comparison with Tukey's test at *p* ≤ 0.05.

Diet type, antibiotic treatment and their interaction had no significant effect on the mean longevity of male or female *B. minax* (figure 1, table 2). Similarly, bacterial supplementation of a sucrose-only diet did not have any effect on the longevity of male and female flies (table 3, *F*_2,6_ = 1.648, *p* = 0.269 and *F*_2,6_ = 3.162, *p* = 0.115, respectively).

**Table 2.** Effects of diet type, antibiotic treatment and their interaction on the longevity of *B. minax*. Separate two-way ANOVAs were run for males and females.

	male longevity		female longevity	
source of variation	*F*_1, 8_	*p*-value	*F*_1,8_	*p*-value
diet	0.91	0.77	2.37	0.16
antibiotic treatment	4.22	0.074	3.51	0.10
diet × antibiotic treatment	1.13	0.32	0.10	0.76

**Table 3.** The effect of diet supplementation with bacteria and antibiotics (10 µg ml^−1^ ciprofloxacin and 200 µg ml^−1^ piperacillin) on different fitness parameters of *B. minax*. SA, sucrose + antibiotics; SAKO, SA + *K. oxytoca*; SACF, SA + *C. freundii*. Rows with the same letters are not significantly different at *p* ≤ 0.05.

	treatments		
	SA	SAKO	SACF
male longevity	49.643 ± 0.41^a^	40.767 ± 0.41^a^	44.167 ± 0.41^a^
female longevity	55.27 ± 3.61^a^	48.43 ± 3.61^a^	61.28 ± 3.61^a^
copulation	0.67 ± 0.58^a^	2.67 ± 0.58^a^	1.67 ± 0.58^a^
fecundity	0	0	0

### 3.2. Number of matings

**Figure 2.**
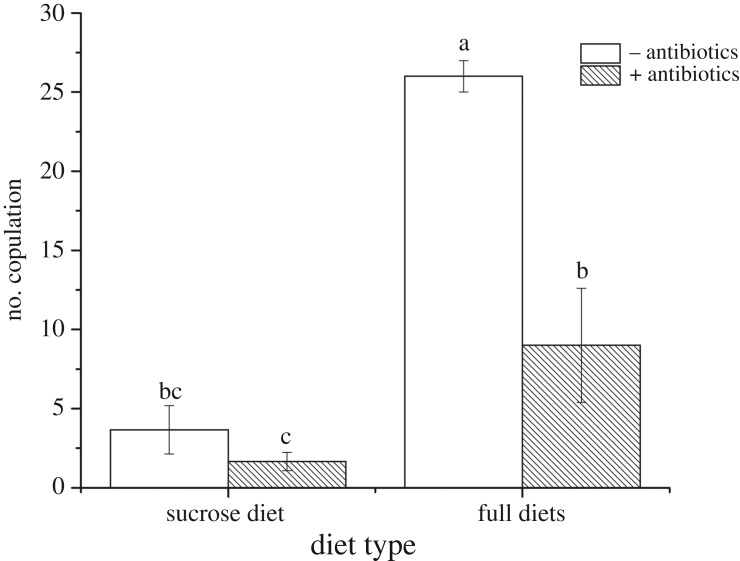
Effect of diet quality and antibiotics (10 µg ml^−1^ ciprofloxacin and 200 µg ml^−1^ piperacillin) on the mean (±s.d.) number of *B. minax* copulations. Unshaded bars are diets with no antibiotics added, shaded bars are diets with antibiotics added. Columns with the same letters are not significantly different after comparison with Tukey's test at *p* ≤ 0.05.

Flies fed sucrose diet achieved significantly fewer copulations than flies fed full diets (*F*_1,8_ = 158.42, *p* < 0.001). Similarly, flies fed with antibiotics achieved significantly fewer copulations than flies without antibiotic treatment (*F*_1,8_ = 64.980, *p* < 0.001) (figure 2). There was a significant interaction between diet and antibiotics (significant *F*_1,8_ = 168.75, *p* < 0.001). The number of copulations achieved by flies on full diets with antibiotics was significantly lower than the number of copulations achieved by flies on full diets without antibiotics. For the sucrose diet, addition of antibiotics to the diet did not significantly reduce (the already low) number of matings (figure 2). Bacterial supplementation to sucrose diets did not significantly alter the number of matings from the sucrose-only diets (table 3, *F*_2.6_ = 4.11, *p* = 0.125).

### 3.3. Female fecundity

**Figure 3.**
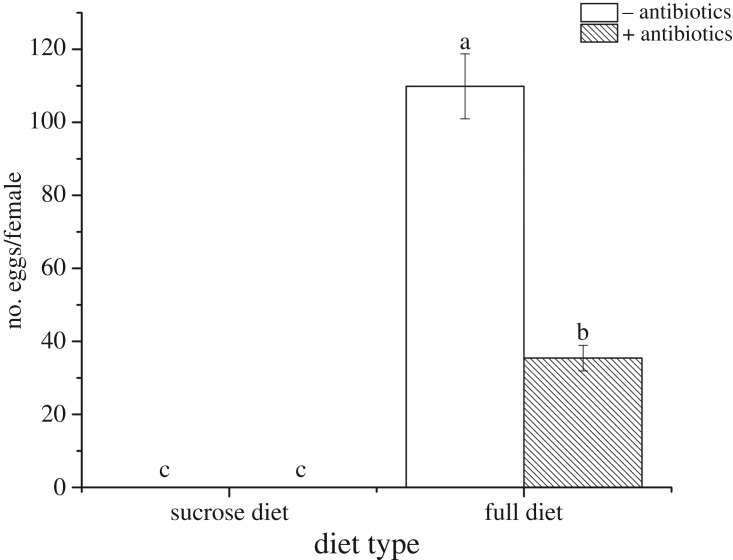
Effect of diet quality and antibiotic treatment on mean (±s.d.) number of eggs laid by *B. minax*. Unshaded bars are diets with no antibiotics added, shaded bars are diets with antibiotics added. Columns with the same letters are not significantly different after comparison Tukey's test at *p* ≤ 0.05.

Diet type dramatically impacted egg production, with flies that fed on a sucrose-only diet, with or without antibiotics, laying no eggs. Similarly, females with bacterial supplementation to sucrose diets did not produce any eggs (table 3). For flies on the full diet, antibiotic treatment significantly reduced the number of eggs laid (*F*_1,4_ = 182.38, *p* < 0.0001) (figure 3).

## 4. Discussion

### 4.1. Summary

Although quantification of bacterial knock-down was not directly estimated after antibiotic treatment, its effect on fitness was seen in the results. The removal of gut bacteria through antibiotic treatment had no effects on the longevity of male and female *B. minax* (figure 1, table 2), but did reduce mating success and egg number. The effects of antibiotic treatments were only significant in the full diets, as the sucrose-only diet was apparently so poor that few matings were observed and no eggs at all were laid (figure 2), even after bacterial supplementation (table 3). Combined, these results strongly infer that bacteria alone are not an adequate source of nitrogen for *B. minax*, but the bacteria do mediate and improve the quality of diet when at least some alternative protein source is available.

### 4.2. Comparison of bacteria effects on fitness of *B. minax* versus studies in other tephritids

The gut of the *B. minax* fly is inhabited by a wide range of symbiotic bacteria [24] (herein referred to as resident bacteria) which enhanced fecundity and copulation rates of flies fed with full diets (data herein and [26]). Limited but similar results have been reported in other tephritids. In *B. oleae*, symbiotic bacteria have been reported to improve on the fecundity of flies fed with sucrose and non-essential amino acids [17] or bird droppings [13]. Similarly, Ben-Yosef *et al*. [30] reported that in the presence of resident bacteria, *C. capitata* fed with full diets had a reduced latency to mate.

While bacterial presence may improve dietary outcomes when amino acids/proteins are part of diet, this does not appear to be the case for carbohydrate diets only. The presence of resident bacteria did not improve any of the measured fitness parameters in sugar-only fed *B. minax* flies. Studies with *B. oleae* [13,17] and *C. capitata* have reported similar findings [30,31]. However, after antibiotics depletion of resident bacteria in *C. capitata*, flies that were fed with sugar diets laid eggs faster than those fed on full diets [30] and increased male longevity [31]. In *Drosophila*, axenic conditions do not lead to a significant increase in lifespan [34], while in the nematode *Caenorhabditis elegans* axenic conditions significantly increased lifespan [35]. From such studies, it is clear that the impact of resident bacteria on host fitness varies depending on the species.

When bacteria were supplemented in the diet of *B. tryoni*, no improvement on fitness was observed [20], a result identical to those was obtained for *B. minax*. However, in *C. capitata*, supplementations with *Pseudomonas* sp. resulted in a decrease in longevity [36] and supplementation with Enterobacteriacea resulted in an increase in mating competiveness [37] and longevity [36]. Similarly, when *C. braaki* and *K. pneumonia* were supplemented in full diet of *B. minax*, there was an increase in mating and fecundity [26]. The conflict between these studies again reinforces the likely unique nature of the bacterial/fruit fly interaction, which appears to vary depending on bacterial species, fly species, other dietary components and the fitness parameter being measured.

### 4.3. Bacteria effects as modified by protein-rich versus protein-poor diets

In many tephritid species, a protein diet is necessary for sexual maturation and oogenesis of the female fly [38,39]. In the absence of a protein diet, *B. minax* were not able to produce eggs, even when their diets were supplemented with bacteria (figure 3). However, when protein was present along with bacteria, there were positive fitness outcomes. Two hypotheses have been postulated to explain how diet and bacteria may work together. The first hypothesis that fruit fly can acquire protein and other nutrients by cultivating and digesting their gut bacteria [40,41], and the presence of additional protein enhances bacterial cultivation. With respect to this, it should be noted that the yeast extract and trypton used in our experiment supports bacterial growth in the laboratory. The second hypothesis proposes that fruit fly gut bacteria use amino acids already present in the diet as building blocks for essential amino acids, which are then used by the fly in their free form or integrated into bacterial protein after their secretion into the gut [13,17]. Because the presence of bacteria in a sucrose-only diet still did not allow the production of eggs by *B. minax*, we can infer that bacteria are not used directly by the fly as protein source, and so discount the first hypothesis. This leaves hypothesis 2 as the most parsimonious explanation: gut bacteria in *B. minax* alter the quality (i.e. type) of amino acid in the fly's diet and this accounts for the significant interaction between bacteria and diet quality.

## Data accessibility

The sequences of *K. oxytoca* and *C. freundii* used in this experiment have been submitted to the NCBI GenBank under the reference number KF145191 and KF145194, respectively.

## References

[1] Douglas A 1998 Nutritional interactions in insect-microbial symbioses: aphids and their symbiotic bacteria *Buchnera*. Annu. Rev. Entomol. 43, 17–37. (10.1146/annurev.ento.43.1.17) 15012383

[2] Tokuda G, Watanabe H, Matsumoto T, Noda H 1997 Cellulose digestion in the wood-eating higher termite, *Nasutitermes takasagoensis* (Shiraki): distribution of cellulases and properties of endo-β-1, 4-gIucanase. Zoolog. Sci. 14, 83–93. (10.2108/zsj.14.83) 9200983

[3] Bourtzis K, Miller TA (eds). 2003 Insect symbiosis. Boca Raton, FL: CRC Press.

[4] Chen R, Wang Z, Chen J, Jiang L-Y, Qiao G-X 2017 Insect-bacteria parallel evolution in multiple-co-obligate-aphid association: a case in Lachninae (Hemiptera: Aphididae). Sci. Rep. 7, 10204 (10.1038/s41598-017-10761-9) 28860659 PMC5579299

[5] Margulis L, Fester R (eds). 1991 Symbiosis as a source of evolutionary innovation: speciation and morphogenesis. Cambridge, MA: MIT Press. 11538111

[6] Margulis L, Sagan D (eds). 2008 Acquiring genomes: a theory of the origins of species. New York, NY: Basic Books.

[7] Gündüz EA, Douglas A 2009 Symbiotic bacteria enable insect to use a nutritionally inadequate diet. Proc. R. Soc. B 276, 987–991. (10.1098/rspb.2008.1476) PMC2664372 19129128

[8] Douglas AE 2015 Multiorganismal insects: diversity and function of resident microorganisms. Annu. Rev. Entomol. 60, 17–34. (10.1146/annurev-ento-010814-020822) 25341109 PMC4465791

[9] Engel P, Moran NA 2013 The gut microbiota of insects–diversity in structure and function. FEMS Microbiol. Rev. 37, 699–735. (10.1111/1574-6976.12025) 23692388

[10] Su Q, Xie W, Wang S, Wu Q, Liu B, Fang Y, Xu B, Zhang Y 2014 The endosymbiont *Hamiltonella* increases the growth rate of its host *Bemisia tabaci* during periods of nutritional stress. PLoS ONE 9, e89002 (10.1371/journal.pone.0089002) 24558462 PMC3928334

[11] Jurkevitch E 2011 Riding the Trojan horse: combating pest insects with their own symbionts. Microb. Biotechnol. 4, 620–627. (10.1111/j.1751-7915.2011.00249.x) 21338477 PMC3819011

[12] Lauzon CR 2003 Symbiotic relationships of tephritids, vol. 1. Boca Raton, FL: CRC Press.

[13] Ben-Yosef M, Pasternak Z, Jurkevitch E, Yuval B 2014 Symbiotic bacteria enable olive flies (*Bactrocera oleae*) to exploit intractable sources of nitrogen. J. Evol. Biol. 27, 2695–2705. (10.1111/jeb.12527) 25403559

[14] Hagen KS 1966 Dependence of the olive fly, *Dacus oleae*, larvae on symbiosis with *Pseudomonas savastanoi* for the utilization of olive. Nature 209, 423–424. (10.1038/209423a0)

[15] Tsiropoulos G 1984 Amino-acid synthesis in adult *Dacus oleae* (Gmelin)(Diptera Tephritidae) determined with [U-^14^C] glucose. Arch. Int. Physiol. Bochim. 92, 313–316. (10.3109/13813458409071172) 6085250

[16] Miyazaki S, Boush GM, Baerwald RJ 1968 Amino acid synthesis by *Pseudomonas melophthora*, bacterial symbiote of *Rhagoletis pomonella* (Diptera). J. Insect. Physiol. 14, 513–518. (10.1016/0022-1910(68)90066-8) 5649231

[17] Ben-Yosef M, Aharon Y, Jurkevitch E, Yuval B 2010 Give us the tools and we will do the job: symbiotic bacteria affect olive fly fitness in a diet-dependent fashion. Proc. R. Soc. B 277, 1545–1552. (10.1098/rspb.2009.2102) PMC2871834 20071385

[18] Behar A, Yuval B, Jurkevitch E 2005 Enterobacteria-mediated nitrogen fixation in natural populations of the fruit fly *Ceratitis capitata*. Mol. Ecol. 14, 2637–2643. (10.1111/j.1365-294X.2005.02615.x) 16029466

[19] Drew R, Courtice A, Teakle D 1983 Bacteria as a natural source of food for adult fruit flies (Diptera: Tephritidae). Oecologia 60, 279–284. (10.1007/BF00376839) 28310683

[20] Meats A, Streamer K, Gilchrist A 2009 Bacteria as food had no effect on fecundity during domestication of the fruit fly, *Bactrocera tryoni*. J. Appl. Entomol. 133, 633–639. (10.1111/j.1439-0418.2009.01420.x)

[21] Dong Y-C, Wang Z-J, Clarke AR, Pereira R, Desneux N, Niu C-Y 2013 Pupal diapause development and termination is driven by low temperature chilling in *Bactrocera minax*. J. Pest Sci. 86, 429–436. (10.1007/s10340-013-0493-y)

[22] Dorji C, Clarke AR, Drew RAI, Fletcher BS, Loday P, Mahat K, Raghu S, Romig MC 2006 Seasonal phenology of *Bactrocera minax* (Diptera: Tephritidae) in western Bhutan. Bull. Entomol. Res. 96, 531–538. 17092364

[23] Liu H, Jiang G, Zhang Y, Chen F, Li X, Yue J, Ran C, Zhao Z 2015 Effect of six insecticides on three populations of *Bactrocera* (*Tetradacus*) *minax* (Diptera: Tephritidae). Curr. Pharm. Biotechnol. 16, 77–83. (10.2174/138920101601150105105751) 25564253

[24] Wang A, Yao Z, Zheng W, Zhang H 2014 Bacterial communities in the gut and reproductive organs of *Bactrocera minax* (Diptera: Tephritidae) based on 454 pyrosequencing. PLoS ONE 9, e106988 (10.1371/journal.pone.0106988) 25215866 PMC4162550

[25] Wang X-L, Zhang R-J 2009 Review on biology, ecology and control of *Bactrocera* (*Tetradacus*) *minax* Enderlein. J. Environ. Entomol. 31, 73–79.

[26] Rashid MA, Andongma AA, Dong Y-C, Ren X-M, Niu C-Y 2018 Effect of gut bacteria on fitness of the Chinese citrus fly, *Bactrocera minax* (Diptera: Tephritidae). Symbiosis 1–7. (10.1007/s13199-018-0537-4)

[27] Aharon Y, Pasternak Z, Ben-Yosef M, Behar A, Lauzon C, Yuval B, Jurkevitch E 2013 Phylogenetic, metabolic, and taxonomic diversities shape Mediterranean fruit fly microbiotas during ontogeny. Appl. Environ. Microbiol. 79, 303–313. (10.1128/AEM.02761-12) 23104413 PMC3536086

[28] Andongma AA, Wan L, Dong Y-C, Desneux N, White JA, Niu C-Y 2015 Pyrosequencing reveals a shift in symbiotic bacteria populations across life stages of *Bactrocera dorsalis*. Sci. Rep. 5, 9470 (10.1038/srep09470) 25822599 PMC5380164

[29] Neto S et al. 2012 Mass-rearing of Mediterranean fruit fly using low-cost yeast products produced in Brazil. Sci. Agricola 69, 364–369. (10.1590/S0103-90162012000600004)

[30] Ben-Yosef M, Jurkevitch E, Yuval B 2008 Effect of bacteria on nutritional status and reproductive success of the Mediterranean fruit fly *Ceratitis capitata*. Physiol. Entomol. 33, 145–154. (10.1111/j.1365-3032.2008.00617.x)

[31] Ben-Yosef M, Behar A, Jurkevitch E, Yuval B 2008 Bacteria–diet interactions affect longevity in the medfly–*Ceratitis capitata*. J. Appl. Entomol. 132, 690–694. (10.1111/j.1439-0418.2008.01330.x)

[32] Ben-Yosef M, Pasternak Z, Jurkevitch E, Yuval B 2015 Symbiotic bacteria enable olive fly larvae to overcome host defences. R. Soc. open sci. 2, 150170 (10.1098/rsos.150170) 26587275 PMC4632588

[33] Dong Y, Wan L, Pereira R, Desneux N, Niu C 2014 Feeding and mating behaviour of Chinese citrus fly *Bactrocera minax* (Diptera, Tephritidae) in the field. J. Pest Sci. 87, 647–657. (10.1007/s10340-014-0605-3)

[34] Ren C, Webster P, Finkel SE, Tower J 2007 Increased internal and external bacterial load during *Drosophila* aging without life-span trade-off. Cell Metab. 6, 144–152. (10.1016/j.cmet.2007.06.006) 17681150

[35] Houthoofd K, Braeckman BP, Lenaerts I, Brys K, De Vreese A, Van Eygen S, Vanfleteren JR 2002 Axenic growth up-regulates mass-specific metabolic rate, stress resistance, and extends life span in *Caenorhabditis elegans*. Exp. Gerontol. 37, 1371–1378. (10.1016/S0531-5565(02)00173-0) 12559406

[36] Behar A, Yuval B, Jurkevitch E 2008 Gut bacterial communities in the Mediterranean fruit fly (*Ceratitis capitata*) and their impact on host longevity. J. Insect. Physiol. 54, 1377–1383. (10.1016/j.jinsphys.2008.07.011) 18706909

[37] Ben Ami E, Yuval B, Jurkevitch E 2010 Manipulation of the microbiota of mass-reared Mediterranean fruit flies *Ceratitis capitata* (Diptera: Tephritidae) improves sterile male sexual performance. Int. Soc. Microb. Ecol. J. 4, 28–37. 10.1038/ismej.2009.82 19617877

[38] Drew R, Yuval B (eds). 1999 The evolution of fruit fly feeding behavior. Boca Raton, FL: CRC Press.

[39] Meats A, Leighton S 2004 Protein consumption by mated, unmated, sterile and fertile adults of the Queensland fruit fly, *Bactrocera tryoni* and its relation to egg production. Physiol. Entomol. 29, 176–182. (10.1111/j.1365-3032.2004.00383.x)

[40] Panizzi AR, Parra JR (eds). 2012 Insect bioecology and nutrition for integrated pest management. Boca Raton, FL: CRC Press.

[41] Lemos F, Terra WR 1991 Digestion of bacteria and the role of midgut lysozyme in some insect larvae. Comp. Biochem. Physiol. B 100, 265–268. (10.1016/0305-0491(91)90372-K) 1799969

